# Influence of acculturation and cultural values on the self-reference effect

**DOI:** 10.1038/s41598-023-46210-z

**Published:** 2024-01-18

**Authors:** Ashley N. Gilliam, Angela Gutchess

**Affiliations:** Department of Psychology, Brandeis University, 415 South St., Waltham, MA 02543 USA

**Keywords:** Human behaviour, Cognitive neuroscience

## Abstract

Cultural milieu can influence the way information is processed and what strategies are employed to deal with ever-changing environments. This study assessed whether acculturation and cultural values of East Asians can affect memory, with a specific focus on the self-reference effect in Chinese international students. Participants encoded and retrieved adjectives, with some trials relating the words to the self (i.e., the self-referencing task), another person, or a control condition; participants also completed questionnaires assessing cultural adaptation and self-construal. Results did not show a relationship between acculturation orientation and self-construal and the magnitude of the self-reference effect in memory, defined as better memory for adjectives encoded related to the self compared to those related to close others, in this sample of Chinese international students. Future research should explore effects of acculturation over time, incorporating more heterogeneous samples and sensitive neural measures.

## Introduction

Research on cross-cultural perception and experience-dependent plasticity has shown that cultural milieu can influence the way information is processed and what strategies are employed to attend to and manage ever-changing environments^1–4^. Previous cross-cultural research has focused primarily on comparing East versus West. Considering today’s increasingly globalized society, it is important to understand how competing cultural perspectives affect behavior. These influences include acculturation, defined here as a cultural change in individuals resulting from continuous first-hand contact between two distinct cultural groups, a definition consistent with “acculturative strategy” as defined by Berry^5^. Because immigrants to a new country are fully immersed in a new cultural context, studying this group offers an ideal opportunity to assess how a novel cultural milieu influences information processing. However, there are few studies that examine the influence of acculturation on memory. In this study, we examined the influence of acculturation and cultural values on the self-reference effect in memory in immigrants to the US from mainland China in order to explore whether individual difference factors might impact the size of the self-reference effect in memory.

The self-reference effect occurs when information related to the self is remembered better than if it were related to something or someone else, even a close other like a mother^6–8^. This has primarily been demonstrated for Western cultures and differs in East Asian cultures such that the distinction between self and close other is reduced compared to Western cultures^9–13^. In one study, Chinese participants recruited the medial prefrontal cortex (MPFC), an area shown in research to contribute to the sense of self and to be important for the retrieval of self-knowledge, during encoding of both the self and mother, perhaps reflecting their collective identities^13^. In contrast, Westerners only recruited the MPFC during self-referencing^13,14^.

Previous work, however, also suggests that individual differences could be important to consider within East Asian samples. For Taiwanese participants, cross-cultural differences in the self-reference effect were limited to older adults^12^; younger Taiwanese and American adults exhibited similar self-reference effects. It is thus vital to understand the contribution of individual differences to self-reference effects in memory.

One individual difference investigated in this study is self concept. Because identity, self-concept, and culture are intimately linked, cross-cultural differences in the self-reference effect may reflect individual differences in self-construal. In an independent view, the self is emphasized as being unique and distinct from others; in an interdependent view of the self, connectedness and relationships with others are emphasized^15^. Self-construal has been shown to vary cross-culturally, such that Westerners are more independent and Easterners are more interdependent^16^, but it can also vary within a culture and across sub-cultures^17–20^. However, within-group differences in the self-reference effect in memory should be further examined in relation to self-construal.

In addition to self-construal, we investigated how individual differences in acculturation impact the self-reference effect. In previous work examining cultural change and individual variation in self-referencing, Huff and colleagues^21^ found in a sample of Asian Americans who had lived in both the US and an Asian country that the way in which one conceptualized of their bicultural identity—as blended into a single identity or alternating between two distinct identities—affected patterns of brain activity during self and other person judgments in regions associated with self-referencing. Another way of investigating acculturative influences in past work has been through priming. For example, Chiao and colleagues^22^ found that priming a collectivistic or individualistic self construal influenced MPFC and PCC activity during self-judgements. In one of the only studies to investigate acculturative effects on self-referencing over time, as participants from China acculturated to the US, the pattern of MPFC engagement for self vs. mother judgments diverged based on individuals’ cultural style. The difference in MPFC activity between mother and the self was maintained in those participants who became less Eastern over a 6-month period whereas activity for the mother and self conditions became more similar in those participants who became more Eastern over time^23^. The present study will extend this past research on acculturation and bicultural influences into the domain of memory.

Our approach to studying acculturation was influenced by Berry’s^5^ model. That is, acculturation strategy, or how one approaches and adapts to the process of cultural change, can be understood as a construct that can vary across time points and contexts^5,24^. Generally, immigrant individuals can be classified into four groups based on acculturation attitudes or strategies: integration, separation, assimilation, or marginalization. Previous research defines these strategies in terms of how important immigrants perceive it to be to 1) preserve their native culture and/or 2) create and maintain relationships with cultural outsiders^25^. This project used this framework for acculturation orientation as well as the duration of time in the US to define the construct of acculturation. In our study, the two subscales were used as continuous measures, rather than categorizing individuals into one of the four groups. Although this operationalization of acculturation does not allow for comparison of all four acculturation styles or groups, the approach maximizes power as one of the first study to examine the impact of acculturation on self-referencing in memory. Both attitudes towards one’s host and home cultures, in addition to length of exposure to a host culture, may influence one’s self-concept and how one internalizes their cultural values and norms. Thus, we assessed potential contributions of both factors—attitudes towards one’s host and home cultures—to the self-reference effect in memory.

### Predictions

We hypothesized that acculturation orientation and cultural values would influence the memory strategies used by Chinese immigrants to the US. Hypothesis one predicted that individuals who had a stronger motivation to form/maintain *host* culture relationships (i.e., with American culture) would exhibit a larger self-reference effect in memory, relative to those individuals who had a stronger motivation to maintain their native identity/culture (i.e., Chinese culture). The second piece to this explanatory framework was the influence of independence/interdependence on memory strategies. Hypothesis two predicted that higher levels of *independence* will predict a larger self-reference effect in memory, relative to those individuals who are more interdependent. Although hypotheses 1 & 2 focused on the self condition, it is possible that acculturation and cultural values could instead impact the other person condition. For this reason, we also tested the influence of acculturation and self-construal on memory for a close other person condition relative to a control condition. We predicted larger other person effects for participants with stronger host than home acculturation orientations (hypothesis 3) and who are more independent than interdependent (hypothesis 4).

## Methods

### Ethics approval

This study was approved by Brandeis’ Institutional Review Board under protocol 19122r. All procedures performed in this study involving human participants were in accordance with the ethical standards of the institutional research committee and with the 1964 Helsinki Declaration and its later amendments or comparable ethical standards.

### Participants

98 Chinese participants were recruited for this study between November 25, 2019 and December 10, 2021. The final sample size was 92 after 6 participants were removed for invalid data or missing data. For questionnaires, data were first examined for missing values and invalid scores. Participants with more than half of survey items missing, who had never lived in the US (i.e., those who had not spent any number of months in the US due to disruptions from the COVID pandemic), or who had an average memory performance across all conditions (as measured by hit minus false alarm rate) below chance were removed.

Participants were from mainland China, had lived in the US for fewer than five years (to focus on early impacts of acculturative experiences in the US), were non-native speakers of English, and between the ages of 18 and 35. They were recruited via online sources, including a Brandeis Psychology participant pool offering course credit. All participants were Brandeis international students. 38 participants were recruited and run in-person prior to COVID-19. All others were run online during the COVID-19 pandemic after March 2020. All participants followed the same basic procedure. English fluency was required because the consent form and some demographic questions were presented in English. Demographics of participants can be seen in Table 1. Responses regarding the number of their friends who are Chinese suggest our sample is a rather insular community of international students with 90% of participants reporting all or most of their close friends are Chinese.

**Table 1:** Demographics of participants; as a percentage of sample or the mean (SD).

Variable	N = 92
**Age**	19.86 (1.89)
**Gender**	
Female	65%
Male	32%
Other	3%
**Race**	
Asian	100%
**Education**	
High school graduate	13%
Some college	67%
2 -year degree	1%
4-year degree	16%
Professional degree	2%
**Close friends Chinese**	
A few of them	4%
About half of them	5%
Most of them	62%
All of them	28%
**Current Country of residence**	
China	16%
USA	84%

This table displays the age, gender, race, education, proportion of close friends who are Chinese, and current country of residence for the final sample of 92 Chinese international students. Percentages are provided for categorical variables. For continuous variables, they are presented in "mean (standard deviation)" format.

### Materials

Words for the experimental task were selected from adjectives from Anderson^28^, such as “wealthy” or “nasty”, that had been translated into Mandarin and used in Chinese samples previously^26,27^. The positivity of adjective word items was controlled for by sorting words into ‘bad’, ‘good’, and ‘neutral’ categories based on likability ratings from American subjects by Anderson^28^ and East Asian subjects by Wang and Tue^27^. These words were divided into four counterbalanced lists with equivalent average likability ratings. The lists were assigned to the four different conditions (three “old” conditions, and “new” words presented only at retrieval) in a counterbalanced manner so that the words were assigned to each condition across participants.

All experimental materials were presented in simplified Mandarin. Two native Taiwanese who were fluent in simplified and traditional Mandarin and English and two native mainland Chinese who were fluent in simplified Mandarin and English provided collaborative translations of all materials into simplified Mandarin and edited wordings identified by pilot participants as inaccurate or awkward simplified Mandarin translations. Task instructions were translated to simplified Mandarin from English. For questionnaires, some materials had pre-existing simplified Mandarin translations that were evaluated for accuracy while others were traditional Mandarin translations that were translated to simplified Mandarin. Translators reviewed each other’s translations for accuracy until consensus was reached that no further changes were needed. The same translators were asked to review any changes made to materials when adapting to an online format for data collection during COVID-19 (e.g., removing mentions of the experimenter in the room). The primary task and questionnaires were presented in Mandarin in order to avoid priming American values through the English language.

### Procedure

**Figure 1:**
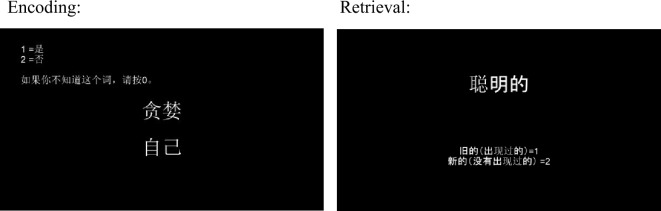
Experimental task example. Figure portions left and right are screenshots from encoding and retrieval sections of the experiment via PsychoPy.

The entire experimental procedure, including the memory task and questionnaires, took approximately 45 min to complete. Participants first signed an informed consent form, and then received instructions and practice on the main experimental task (see Fig. 1 for a visual example of the task). Prior to beginning the encoding task, participants were asked to choose a “close other” (one person) and “farm animal” (one category like “pigs” or “cows” in general) to think of throughout the duration of the experiment (as in^12^). During encoding, participants studied 54 to 55 trait-based words (depending on word lists), presented in Simplified Mandarin on a computer screen. For each trial, participants pressed a key (1 for “yes” or 2 for “no”) to indicate whether the word described the target well. Word items were presented in subject-specific random order for 7 s each at encoding with 250 ms intervals between targets. There were 18–19 trials for each of three conditions—self, close other, and farm animal—and trials were presented in a unique order for each participant. Additionally, there were 9 practice trials (3 trials for each condition) before beginning encoding to allow participants to adjust to the quick response time of the task. Farm animal was used as a control condition that supported semantic judgments (i.e., trait judgments can be made about animals) and in place of the “distant other” that has been used in some studies. This decision reflected the difficulty in selecting an appropriate target across cultural contexts and individual variation in political beliefs and knowledge that allowed for the target to be known and evaluated (e.g., liked) similarly across participants. Previous work has shown that category norms for farm animals were equivalent between cultures^29^, which supported the selection of “farm animal” as an appropriate control condition.

Next was a 10-min retention interval during which time there was a break. The task was programmed so that instructions would not progress until 10 min had passed (with a visual countdown clock on the screen the entire period). Thus, online sessions could be completed independently without an experimenter present. Participants performed the retrieval task for a total of 73 adjectives: 18–19 new words not studied previously and 54–55 were old previously studied words, with 18–19 words per reference condition (self, close other, and farm animal). During the retrieval test, participants viewed an adjective on the screen and then decided whether each item was “old” (previously studied) or “new” (not studied previously). The task was self-paced; once participants responded with a keypress, a blank screen appeared for 250 ms before the program advanced to the next trial.

Participants then completed a Qualtrics survey. This survey included general demographics measures presented in Simplified Mandarin that allowed our samples to be characterized and compared on individual differences of interest. Demographic questions concerned gender, ethnicity, life history of migration, views of the self, etc. Primary predictors of memory performance included 1) the acculturation orientation scale (AOS;^25^) to assess individuals’ relationships to their culture of origin and relationship to the culture of contact, and 2) the Singelis Self-Construal Scale (SCS;^15^) to measure independence and interdependence. Both of these scales used 1–7 bidimensional Likert scales to indicate disagreement to agreement. Sample reliability for the AOS and Singelis Self-Construal subscales were adequate (Independence α = 0.65, Interdependence α = 0.78, AOS Home α = 0.81, AOS Host α = 0.81).

In order to characterize the sample, participants completed additional measures of factors that could impact self-reference and memory performance: the Center for Epidemiologic Studies Depression Scale (CES-D;^30^), which has a 1–4 Likert scale from rarely to most or all of the time and has a total that can range from 20 to 80, and 2) the Psychological Adaptation Scale (PAS;^25^), which uses a 1–7 scale (from never to always). These were used to assess negative emotions related to the immigration process that could impact memory, including items concerning feeling strain from the effort to adapt, missing friends and family back home, and feeling anxious about meeting local people. See Table 2 for descriptive information for questionnaires included in analyses, and supplemental materials for additional measures collected but not used in primary analyses.

**Table 2:** Descriptives and correlations among model variables.

Variable	Mean (SD)	1	2	3	4	5	6	7
1. AOS Host	4.70 (1.09)		0.14	0.25*	0.25*	− 0.14	0.10	− 0.04
2. AOS Home	4.97 (1.23)			− 0.06	0.40***	− 0.29*	0.13	− 0.51***
3. Independence	4.60 (0.61)				− 0.02	0.03	− 0.09	0.16
4. Interdependence	4.50 (0.71)					− 0.02	0.06	− 0.44***
5. Time in the U.S. (months)	12.27 (15.74)						− 0.28*	0.24*
6. CES-D total score	28.14 (15.20)							− 0.30**
7. PAS average score	4.62 (0.93)							

*p < .05, **p < .01, ***p < .001.

This table displays the mean and standard deviation of predictors, covariates, and other exploratory scales that related to the construct of acculturation, self-construal, and mental health. It also displays the correlation between each of these variables.

### Scoring and data analysis

Memory performance was scored as the proportion of correct responses to old items, or hits, minus the number of new items mistakenly remembered as being seen before, or false alarms. The false alarm (FA) rate was the same for all conditions, as there was only one pool of “new” items rather than being specific to each condition.

For all linear regressions, memory performance was quantified using only the hit rate, or correct recognitions of an old item, for each reference condition. This is because the false alarm rate is the same for each condition. A difference score was calculated for self minus close other to address hypotheses one and two. Additionally, a difference score was calculated for close other minus farm animal to examine if any potential relationships were driven by the close other condition, rather than the self condition, to test hypotheses three and four. Difference scores were also calculated for predictors of interest, such that self-construal was operationalized as independence minus interdependence (Adjusted Independence Score), as has been done in prior studies^31,32^. Similarly, acculturation orientation was operationalized as host minus home (Adjusted Host Acculturation Score). Using difference scores allowed for a relative measure of an individual’s tendency towards one style or another, collapsing scores for related constructs into a single measure and minimizing the influence of response bias that could impact the interpretation of scores across individuals.

A one-way ANOVA and Tukey HSD comparisons were used to examine the effect of condition on memory performance (measured by hits minus false alarms). Regressions were then used to analyze the influence of individual differences with two different models for each outcome—one for interdependence/independence and one for acculturation orientation. Separate models were used for acculturation and self-construal as the scales are weakly to moderately correlated (see Table 2). Assumptions, such as homogeneity of variance, were examined with graphs. Exploratory analysis considered the effects of time spent in the United States on memory performance using linear regressions. Covariates for regression models included the Center for Epidemiologic Studies Depression Scale (CES-D) and the Psychological Adaptation Scale (PAS). According to an a priori power analysis, to test our hypotheses using linear regressions with all covariates and predictors included with a power of 0.8 and assuming a medium effect size, we would need a sample of at least 76 participants. We planned to sample to the end of the semester once we reached this sample size, in order to collect data from at least that many participants and account for the possibility of some unusable data.

## Results

### Self-reference memory performance

**Figure 2:**
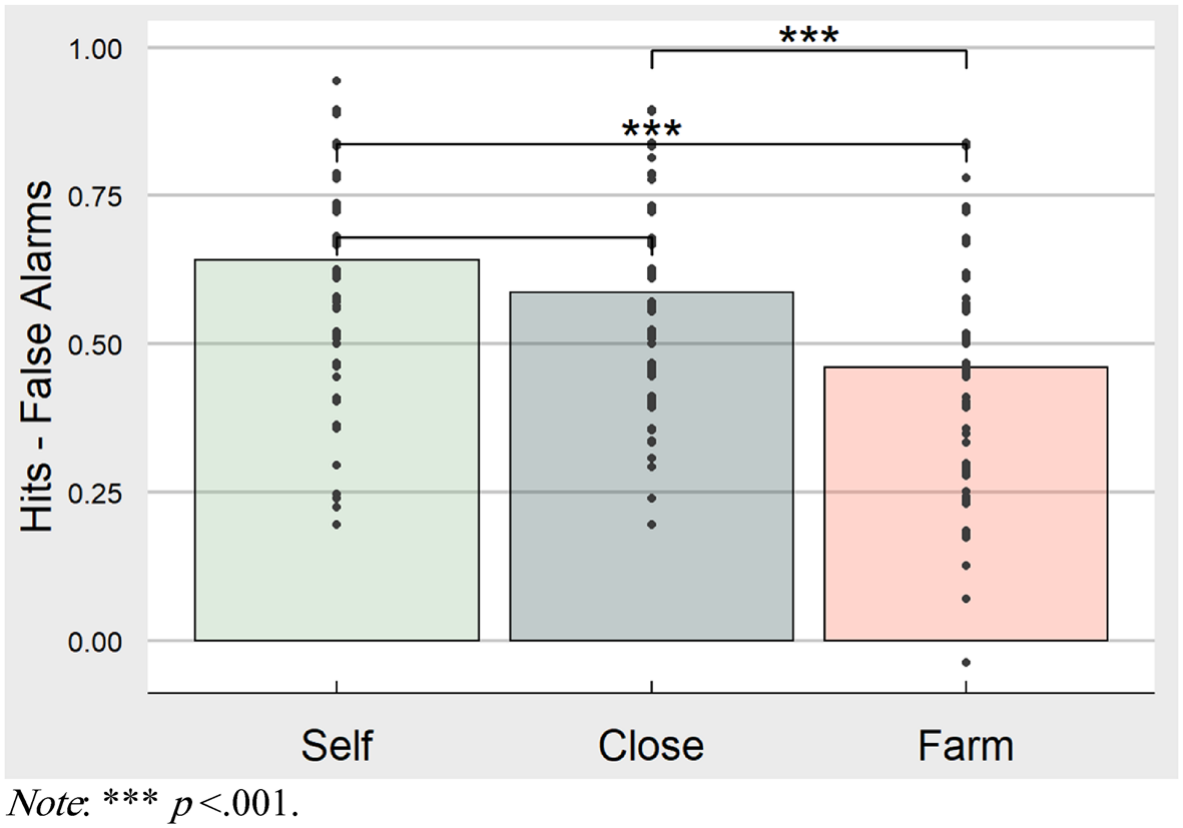
Bar graph of memory performance by condition showing pairwise comparisons. This figure displays the memory performance (number of hits minus number of false alarms) for each reference condition. Sample variability can be seen from individual dots representing each participants' performance (with everyone experiencing every condition). Horizontal lines indicate Tukey HSD pairwise comparisons following an ANOVA and significant differences are denoted with asterisks. The figure was produced in R using ggplot.

To first examine whether there is evidence of a self-reference effect in memory, a one-way ANOVA was used with condition (self, close other, farm animal) as a within-subject variable. Memory performance, measured by hits minus false alarm rate, was the dependent variable. Descriptively, the self condition resulted in the highest level of memory performance (M = 0.64, SD = 0.18), followed by close other (M = 0.59, SD = 0.16), and finally followed by farm animal (M = 0.46, SD = 0.18). Although there was a significant overall effect of condition (F (2, 273) = 26.52, *p* =  < 0.001, η^2^ = 0.16), Tukey HSD comparisons showed that there was not a significant difference between memory performance in the self and close other conditions (*p* = 0.08, *d* = 0.32) whereas comparisons were significant between self and farm animal conditions (*p* < 0.001, *d* = 1.04) and close other and farm animal conditions (*p* < 0.001, *d* = 0.73). See Fig. 2 for a bar graph of task results. For comparison with previous studies, the effect size for the main effect of condition on memory performance in this study with an East Asian sample tested in the US (η^2^ = 0.16) was much smaller than that of previous similar studies using American samples (e.g., η_p_^2^ = 0.53 in Zhang et al.^12^).

### Association between acculturation orientation and the self-reference effect in memory

For hypothesis one, we predicted that a) higher desire to form/maintain host culture relationships versus home culture relationships would predict a larger memory difference score between self and close other conditions (corrected recognition scores for items paired with the self and close other).

To test Hypothesis 1, we examined the effects of acculturation orientation using the Adjusted Host Acculturation Score (based on the Acculturation Orientation Scale; AOS host minus home score) on self-referencing using the following models:

- Y_i_ = α + βAOShost-home_i_ + ε
- Y_i_ = α + βAOShost-home_i_ + βCovariates_i_ + ε

**Figure 3:**
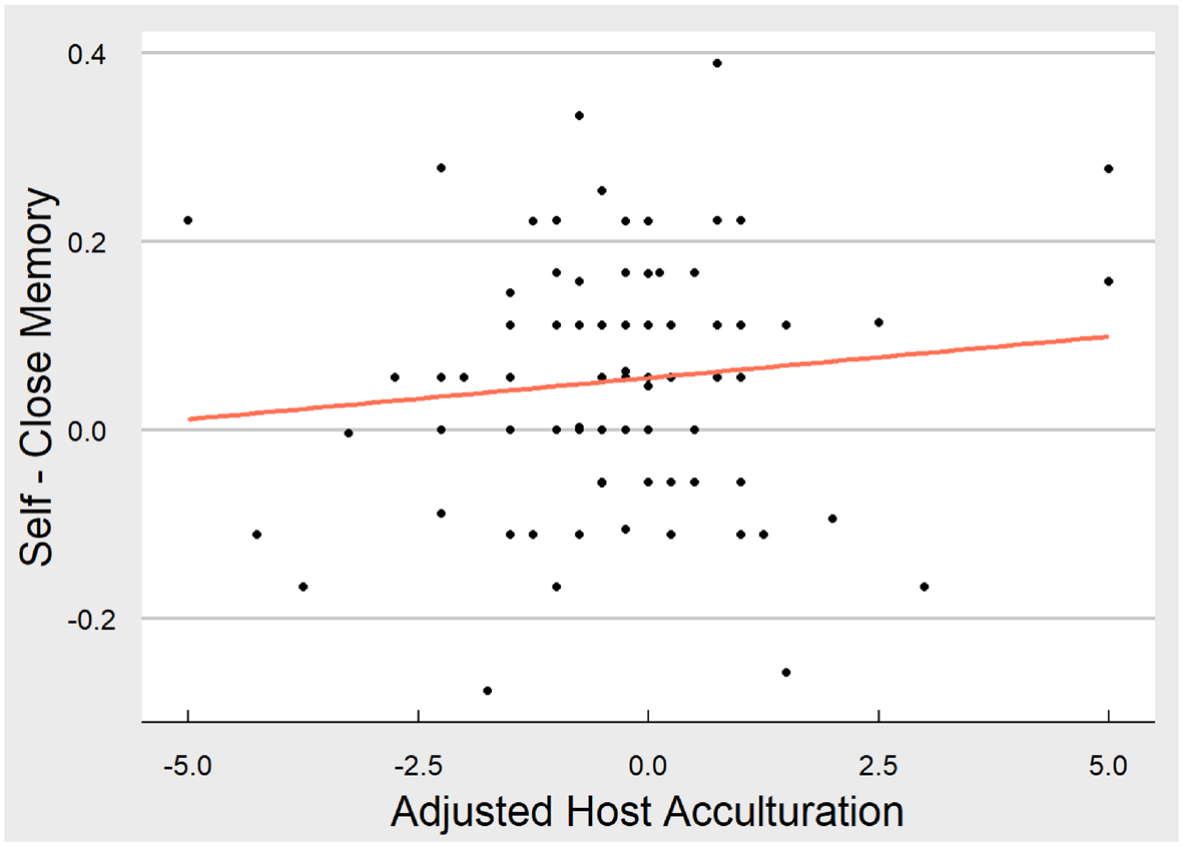
Scatterplot of relationship between SRE and adjusted host acculturation score. This figure displays the linear relationship between memory performance (for self minus close other) and adjusted host acculturation score. Sample variability can be seen from individual dots representing each participant. The figure was produced in R using ggplot.

For model a, the Adjusted Host Acculturation Score did not significantly predict the difference in memory performance between self and close other conditions (βhost-home = 0.11,* t* = 1.00, *p* = 0.32; scatterplot of relationship can be seen in Fig. 3). This continued to be the case when covariates were included in the model (b) (*p* = 0.12). See supplemental materials (Supplement B) for follow-up analyses that include Host and Home scores as separate predictors.

**Figure 4:**
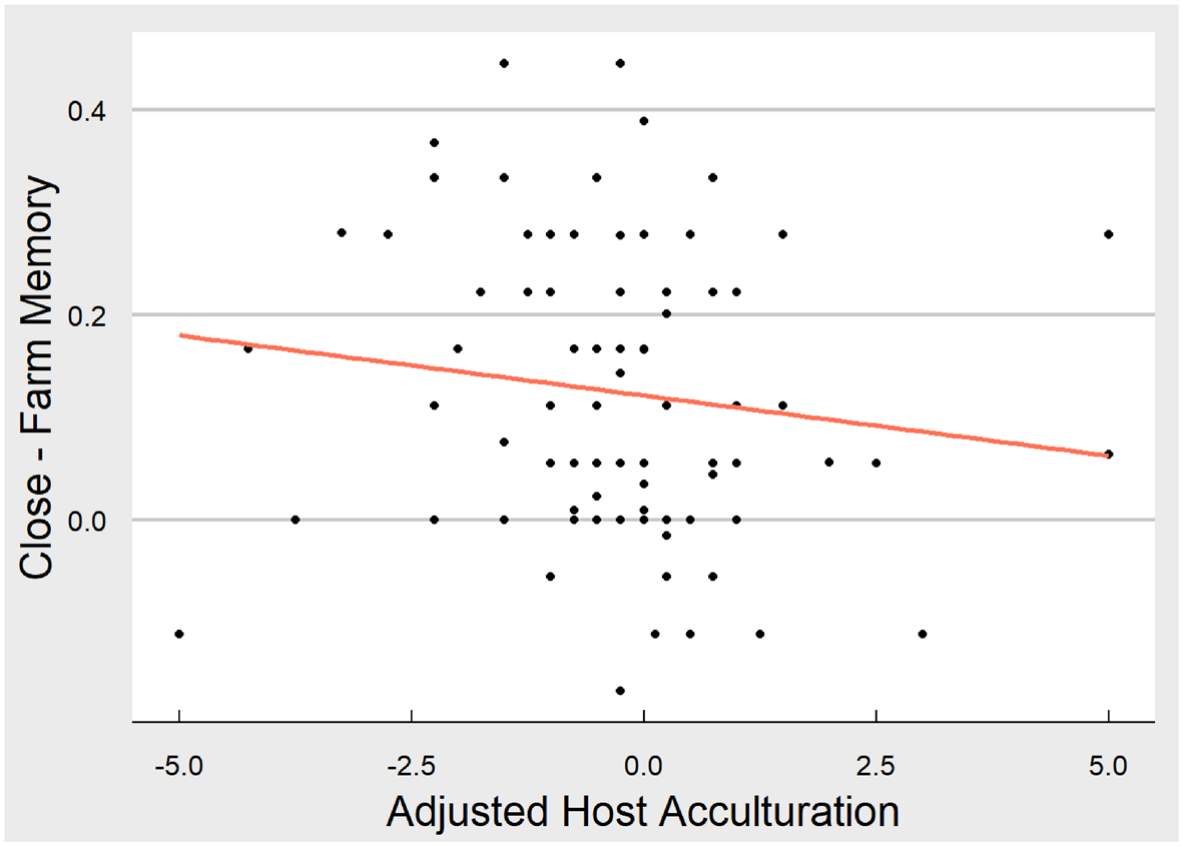
Scatterplot of relationship between memory for close others minus farm animals and adjusted host acculturation score. This figure displays the linear relationship between memory performance (for close other minus farm animal) and adjusted host acculturation score. Sample variability can be seen from individual dots representing each participant. The figure was produced in R using ggplot.

To test hypothesis 3, we examined whether Adjusted Host Acculturation Score predicted memory for close other minus farm animal. This approach allowed us to investigate if any potential differences in self-referencing due to acculturation were driven by memory for close others, rather than memory for the self. In order to address this, the same models a and b were applied with memory for close other minus farm animal as an outcome. For model a, the Adjusted Host Acculturation Score did not significantly predict the difference in memory performance between close other and farm animal conditions (βhost-home = − 0.12, *t* = − 1.19, *p* = 0.24; scatterplot of relationship can be seen in Fig. 4). This continued to be the case with covariates included in the model (b) (*p* = 0.37).

### Association between self-construal and the self-reference effect in memory

For hypothesis two, we predicted that higher independence than interdependence would predict larger memory difference scores between self and close other. To test hypothesis 2, we examined the effects of having a relatively more independent than interdependent (Ind-Inter) self-construal on self-referencing effects in memory using the following models:

(c) Yi = α + βInd-Interi + ε
(d) Yi = α + βInd-Interi + βCovariatesi + ε

**Figure 5:**
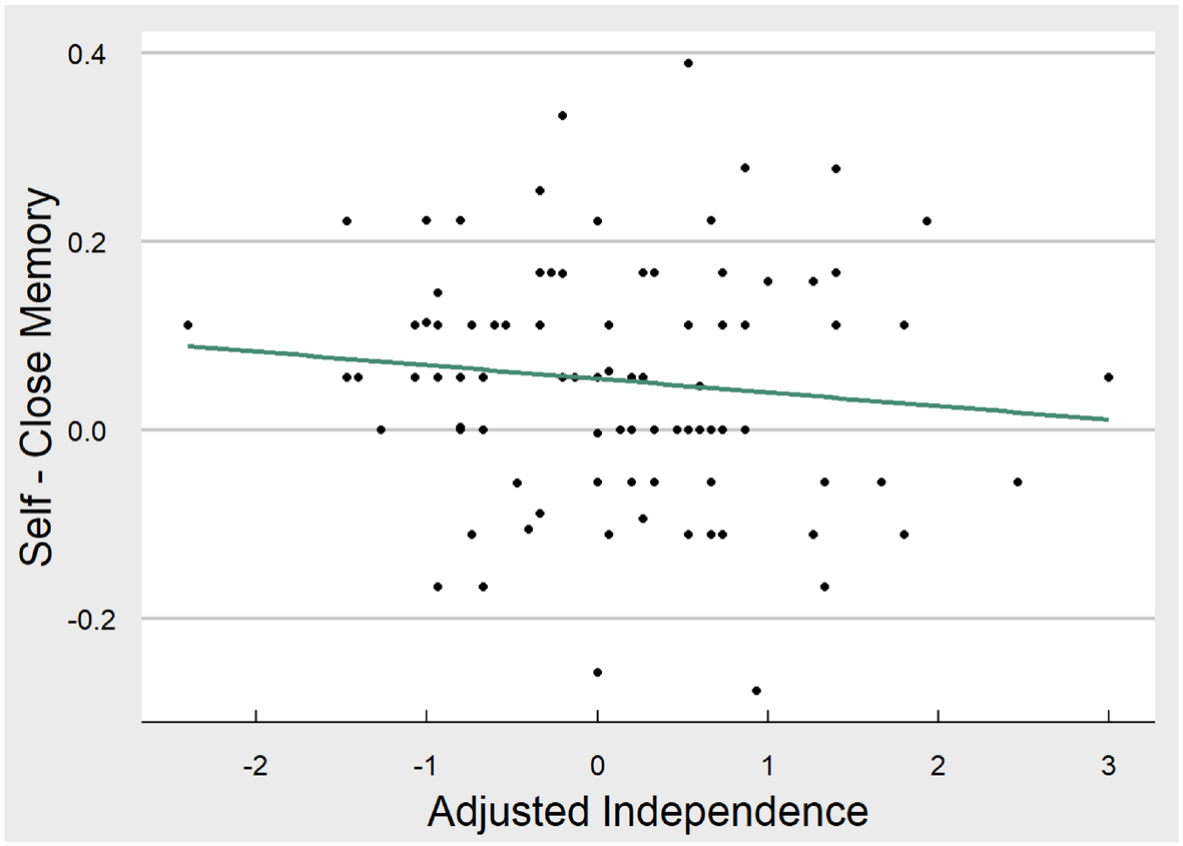
Scatterplot of the relationship between SRE and adjusted independence score. This figure displays the linear relationship between memory performance (for self minus close other) and adjusted independence score. Sample variability can be seen from individual dots representing each participant. The figure was produced in R using ggplot.

For model a, Adjusted Independence Score did not significantly predict the difference in memory performance between self and close other conditions (βind-inter = − 0.11, *t* = − 1.02, *p* = 0.31; scatterplot of relationship can be seen in Fig. 5). This continued to be the case with covariates included in the model (*p* = 0.21).

**Figure 6:**
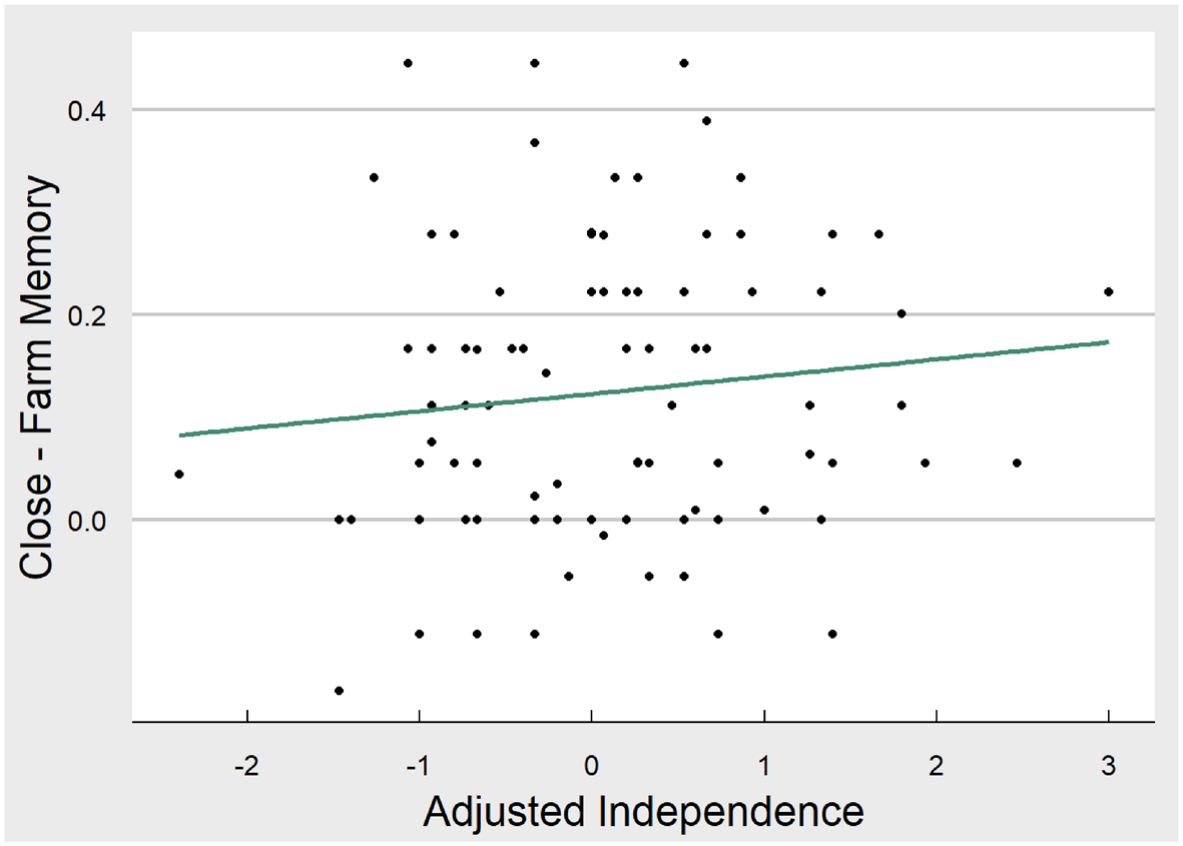
Scatterplot of relationship between memory for close others minus farm animals and adjusted independence score. This figure displays the linear relationship between memory performance (for close other minus farm animal) and adjusted independence score. Sample variability can be seen from individual dots representing each participant. The figure was produced in R using ggplot.

We then tested whether Adjusted Independence Score predicted memory for close other minus farm animal, allowing us to examine if any potential differences in self-referencing were driven by memory for close others, rather than memory for the self (hypothesis 4). In order to address this, the same models c and d were applied with memory for close other minus farm animal as an outcome. For model c, the Adjusted Host Acculturation Score did not significantly predict the difference in memory performance between close other and farm animal conditions (βind-inter = 0.11, *t* = 1.05, *p* = 0.30; scatterplot of relationship can be seen in Fig. 6). This continued to be the case when covariates were included in the model (d) (*p* = 0.28).

To examine the strength of the evidence for the null, Bayes Factors were calculated for each single-predictor model using the *BayesFactor* package in R. There was only anecdotal evidence for the null hypothesis when considering the effect of adjusted host acculturation on self-versus-other memory (BF = 0.34) and close-versus-farm memory (BF = 0.41) as well as the effect of adjusted independence on self-versus-other memory (BF = 0.35) and close-versus farm memory (BF = 0.36), according to guidelines from Andraszewicz and colleagues (2015)^33^.

See the Supplemental Materials for additional analyses considering length of time in the US and comparing the samples as a result of the COVID-19 pandemic.

## Discussion

The self-reference effect is a robust boost in memory for items related to the self, particularly among Westerners. Previous research, however, has indicated that cultures may differ in how much of a memory benefit they receive from a self-referencing strategy. In order to identify culturally-related factors that contribute to the self-reference effect in memory, we tested the influence of acculturative style and cultural values in a sample of international students who recently immigrated to the US from China. We first evaluate evidence for an overall self-reference effect in memory and then discuss the different factors we assessed as related to the self-reference effect in memory.

Our sample of Chinese international students tested in the US descriptively showed the American pattern of performance (self > close > farm animal). However, there was no statistical difference in memory performance between the self and close other conditions in the experiment. This pattern coincides with previous cross-cultural literature examining East Asian memory strategies, which indicates smaller self-reference effects in memory for East Asians than Westerners^9–13^. In fact, effect size estimates were much smaller in the present sample than in a sample of young Americans tested on a similar protocol in our laboratory^12^. This pattern indicates that international students from China can exhibit a reduced self-reference effect compared to Westerners, despite the fact that this sample self-selected to move to the US and was recruited from and, in most cases (84% of sample), tested in the US.

In terms of our hypotheses regarding the roles of acculturation and cultural values, we did not find evidence that either of these factors strongly influenced the magnitude of the self-reference effect. At one level this may suggests that, at least behaviorally, individual differences in these variables are not associated with self-referencing in memory for Chinese international students. Such an interpretation would be consistent with past failures to detect effects of self-construal on memory strategies and performance (as discussed in^34^). Perhaps indicating that memory ability and cognitive strategies such as the self-reference effect are less malleable than more social processes that have shown effects of self-construal (see^16^ for an overview), or that it is more difficult to detect effects of these factors on cognition (e.g., due to stronger effects of memory capacity and attention). It could also suggest that the questionnaires fail to assess the precise cultural values that influence self-referencing. For example, the scales ask about the cultural values in an explicit way, which may be prone to effects of self-reporting and self-awareness, or individuals may even be reflecting comparison to different reference groups (e.g., other international students, American students, acquaintances in China)^35^. Implicit measures of independence-interdependence may be more cross-culturally valid and may lead to more interpretable effects than explicit measures^36^. Additionally, acculturation is a complex construct, composed of a variety of factors including length of time spent in a host culture, language acquisition, media exposure, friendship quantity and quality, cultural and racial identity, discriminatory experience, stress, specific cultural values, and attitudes towards host and home cultures. These aspects may vary in their impact on cognition. Because this was the first study to examine the impact of acculturation on self-referencing, this project focused primarily on one component of acculturation—attitudes towards host and home cultures—as well as self-construal, but future research is needed to explore all these various components and their unique relationships to different domains of cognition. Lastly, future research should consider the potential impact of sensitive periods for acculturation, though evidence for one is mixed^37,38^.

Another question involves our use of measures. Although we focused on acculturation, supplemental materials consider whether other measures of acculturation, identity and cultural values relate to the self-reference effect in memory. In addition, the factor analysis including all of these measures indicates that the constructs we assessed through separate analyses—adjusted host score and adjusted independence—loaded onto the same factor (see Supplement D). Conceptually they are quite distinct constructs, but future research should build on the present results to determine the best ways to assess acculturative factors and their impact on cognition.

It is also possible that we failed to detect potential effects due to limitations that could have affected our ability to detect such effects in the current sample. A more heterogeneous sample may be needed to detect relationships between cultural values, acculturative factors, and a self-referencing memory strategy. Specifically, our sample was highly insular, with 90% of our participants reporting that most or all of their friends were from China. In addition, the majority of participants had been in the US for less than 12 months. Effects on cognition could emerge with more time spent in the US and exposure to its values, compared to a sample that has been in the US for less time. Moreover, because the current study was conducted during the COVID-19 pandemic, the isolation and reduction of in-person cultural contact could have affected the acculturative experiences of our participants (i.e., reduced contact with Americans as activities moved online or students returned to China). In addition, cultural values such as independence-interdependence or tightness-looseness^39^ could have been affected by the COVID-19 pandemic^34,40,41^, potentially leading to greater insularity of international students and thus reducing the size of potential between-subject effects. See Supplemental Materials for exploratory analyses. Recruiting a more heterogeneous sample of Chinese international students, varying on the amount of time in the US and in the amount of contact and connection with Americans, could allow for the detection of more robust effects in future research, particularly if conducted in a more stable cultural environment than 2020–2021.

Future research would benefit from examining the effects of acculturation and self-construal on self-referencing in international students and other immigrants over time. Few cognitive studies have considered transitional identity and values, bicultural identity, or acculturation, especially as a function over time (see^23,42^). It is possible that effects not seen between individuals may become apparent when examining change within an individual over time. Effects in our study at one time point may have been limited due to the homogeneity and insularity of our sample. It is possible that contact with the host culture increases over time, or that the timing of our data collection during the COVID-19 affected acculturation. By examining within-subject change over time, relationships may be more easily detected. There is some prior literature supporting the idea that acculturative influences may not always be evident cross-sectionally but are evident longitudinally^23,42^). Self-construal and acculturation orientation were weakly to moderately correlated in our sample, so examining their relationship to one another over time could help to disentangle their potential effects on cognition. In addition, future work should incorporate neural measures, as previous research has indicated that these may be more sensitive markers of the influence of culture than behavioral measures^43^. Previous work using ERP and fMRI has demonstrated that cultural groups differ in the neural response to self versus close others^40,44,45^.

Our results point to a new avenue of research on self-referencing in memory. Despite the lack of heterogeneity in our sample, the self-reference effect for these young adult Chinese international students was still smaller than that demonstrated in similar research using American samples. This suggests that when increasing the representativeness and generalizability of samples in future research, we may find even greater variation in self-referencing. There are many aspects of cultural experiences and immigration history that could shape cultural values and cognition. Future work could move beyond international college students who have self-selected to immigrate to the United States and recruit a more representative sample of immigrants (e.g., those on work visas, refugees, etc.) By recruiting a more varied sample, factors that could have an impact on values are also more likely to vary, including socioeconomic status, exposure to, use of, or identification with American media, and region of origin within China. Those who have a lower socioeconomic status may display greater interdependence^46^ or have more barriers to integrating with the host culture (e.g., inability to take ESL courses, fewer resources to spend on leisure and social activities), those who use or identify more with American media prior to immigration may acculturate more quickly than those who do not, and those from rice vs wheat growing regions of China may have differing levels of collectivism and interdependence^47^. Bicultural identity integration (BII), how compatible or incongruent two cultural identities are within an individual (e.g., being a part of both Chinese and American cultures) could also be an important factor in determining effects of acculturation on cognition. Rather than showing expected effects of acculturation (e.g., becoming more American with more time in the US), individuals with low BII can exhibit contrast effects, such as responding the opposite of what would be predicted (e.g., exhibiting a more Chinese style rather than an American one) (see^48^). Contrast effects and variability in BII in the sample could account for an anomaly in the relationship amongst variables, in that unadjusted acculturation to host score is moderately positively correlated with both unadjusted independence and unadjusted interdependence. Differing attitudes towards the host culture while adjusting to the US could lead to differing outcomes in terms of self-construal values. However, pattern of correlations could be a simple reflection of response biases (e.g., people who tend to strongly endorse items do so across measures) that have not been corrected in these unadjusted measures. BII was not measured in this study, but will be useful for future work. Moving forward, future research should consider how these factors influence self-concept and cognition, allowing us to better understand the generalizability of cross-cultural influences on the self-reference effect in memory.

### Supplementary Information

Supplementary Information.

## Data Availability

None of the data or materials for the experiment reported here are available currently due to confidentiality and on-going follow-up studies, but the de-identified data are available from the corresponding author on reasonable request. This study was not preregistered.
