# Supplementary material for: Influence of acculturation and cultural values on the self-reference effect
Source: Sci Rep. 2024 Jan 18;14:1624. doi: 10.1038/s41598-023-46210-z (PMC10796948; doi:10.1038/s41598-023-46210-z)
Supplement: Supplementary file 1 — Supplementary Information. [file 41598_2023_46210_MOESM1_ESM.pdf]

Supplemental materials

Influence of Acculturation and Cultural Values on the Self-Reference Effect

Ashley N. Gilliam and Angela Gutchess

Supplement A. *Correlation Matrix of Differenced Scores & Subscales*

To further understand effects and relationships between our variables of interest, we conducted additional correlations. We expanded the correlation table in the main text by creating a correlation matrix of the differenced predictors from the primary model alongside their sub-components.

Supplemental Figure 1. Correlation Differenced & Non-Differenced Variables of Interest
* Denotes significant correlations (p<.05)

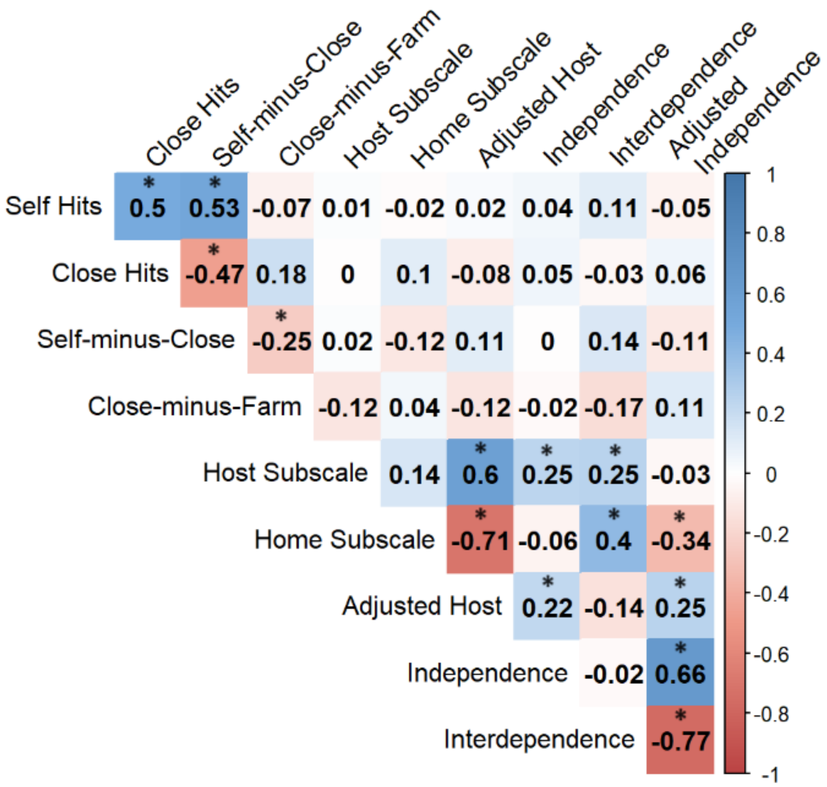

There are moderate positive correlations between the unadjusted host score with unadjusted independence and unadjusted interdependence scores (rs = .25). These could simply reflect response biases (e.g., tendency to endorse items in a similar way across measures). It could also be a result of contrast effects due to differences in Bicultural

Identity Integration (BII). See the main text's discussion section for further discussion of BII.

Supplement B. *Additional Analyses of AOS Host and Home Subscales*

To further understand effects of our predictor variables, we conducted additional linear regression models including scores on Host and Home subscales as separate predictors (as opposed to the difference score). Model structures mirrored that of the main text:

a. $Y_i = \alpha + \beta AOShost_i + \beta AOShome_i + \varepsilon$

b. $Y_i = \alpha + \beta AOShost_i + \beta AOShome_i + \beta Covariates_i + \varepsilon$

Consistent with the primary analyses reported in the main text, no significant associations were found.

There were no significant effects of host acculturation orientation on either self-minus-close memory performance ($p = 0.76$) or close-minus-farm memory performance (0.21) before the addition of covariates in the model. Similarly, there were no significant effects of home acculturation orientation on either self-minus-close memory performance ($p = 0.26$) or close-minus-farm memory performance (0.55) before adding covariates to the model. These effects remained non-significant when adding covariates to models (ps > 0.059)

Supplement C. *Additional Analyses of Time in the US, location of data collection, & COVID*

We were also interested whether time in the US, an objective measure of exposure to the US and potential acculturative influences, was associated with the self-reference effect in memory. When alone in the model, time in the US did not significantly predict the difference in memory performance between self and close other conditions ($\beta = 0.17$, $t = 1.45$, $p = 0.15$). This continued to be the case with covariates included in the model ($p = 0.11$).

Supplemental Figure 2. Scatterplot of the Relationship Between SRE and Time in the U.S.

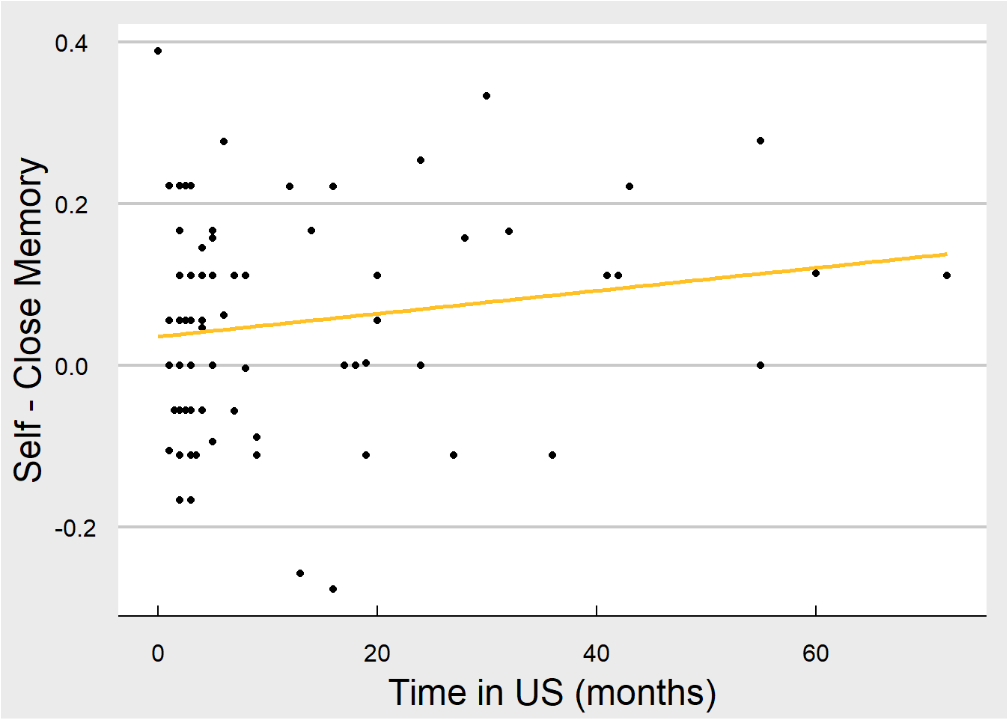

Due to the COVID-19 pandemic, some participants were run in person and some were run online; this also meant that unexpectedly, some participants were tested in the US and some had returned home to China. We tested for the potential influence of COVID-19, current country of residence, and form of data collection, keeping in mind that the three are confounded in this sample. Welch two sample t-tests were run comparing online (n = 53) versus in-person (n = 38) participants in terms of self-versus-close other memory ($t(75.70) = 0.25$, $p = 0.80$), close other-versus-farm animal memory ($t(87.35) = -0.43$, $p = 0.67$), adjusted host acculturation ($t(70.83) = 2.98$, $p < .01$), and adjusted independence ($t(66.19) = 1.25$, $p = 0.21$). Only adjusted host acculturation significantly differed between online and in person groups, such that those run in person prior to COVID's spread were more acculturated to the United States. Welch two sample t-tests were similarly run to examine the difference between participants living in the US and China at time of test in terms of self-versus-close other memory ($t(29.66) = -0.06$, $p = 0.95$), close other-versus-farm animal memory ($t(17.81) = -0.25$, $p = 0.80$), adjusted host acculturation ($t(21.60) = -0.56$, $p = 0.58$), and adjusted independence ($t(30.53) = -2.25$, $p = 0.03$). Only adjusted independence significantly differed between US and China residing samples, such that participants residing in China were on average less independent in terms of self-construal.

The exploratory analyses suggest that adjusted host acculturation differed between online (post-COVID) versus in person (pre-COVID) samples. Additionally, adjusted independence differed between participants residing in the US and China in the

expected direction, such that participants tested in the US were more independent than those tested in China.

Supplement D. *Additional Questionnaires*

Methods. Additional questionnaires were used to characterize the sample, including: 1) the Sociocultural Adaptation Scale, in which participants rated the difficulty of adapting to various aspects of their host culture (SCAS; 1-7 from very difficult to very easy, M = 4.72, SD = 0.96) (Demes & Geeraert, 2014); 2) the perceived cultural distance scale, in which participants rated how similar their host culture is to their home culture on various items (1-7 from very similar to very different, M = 5.16, SD = 0.73) (Demes & Geeraert, 2014); 3) the Schwartz Culture Value Scale (SVS) (-1-7 from opposite to my values to extremely important) to assess cross-cultural values and beliefs (Schwartz, 1992; $M_{achievement} = 1.96$, $SD_{achievement} = 1.46$; $M_{tradition} = -0.29$, $SD_{tradition} = 1.31$; $M_{conformity} = 1.69$, $SD_{conformity} = 1.27$; $M_{self\text{-}direction} = 2.23$, $SD_{self\text{-}direction} = 1.32$).

Exploratory Results. To assess the potential impact of these factors on self-referencing in memory, we first conducted an overall correlation analysis to evaluate how the measures relate to each other. Many of these scales were added to the study during the data collection process (e.g., colleagues suggested additional variables of potential interest), so this analysis could only be conducted on the subsample of 39 participants with complete data. Correlation results are presented below as a matrix.

Supplemental Figure 3. Correlation Matrix All Potential Acculturative Factors (n = 39).
* denotes significant correlations ($p<.05$).

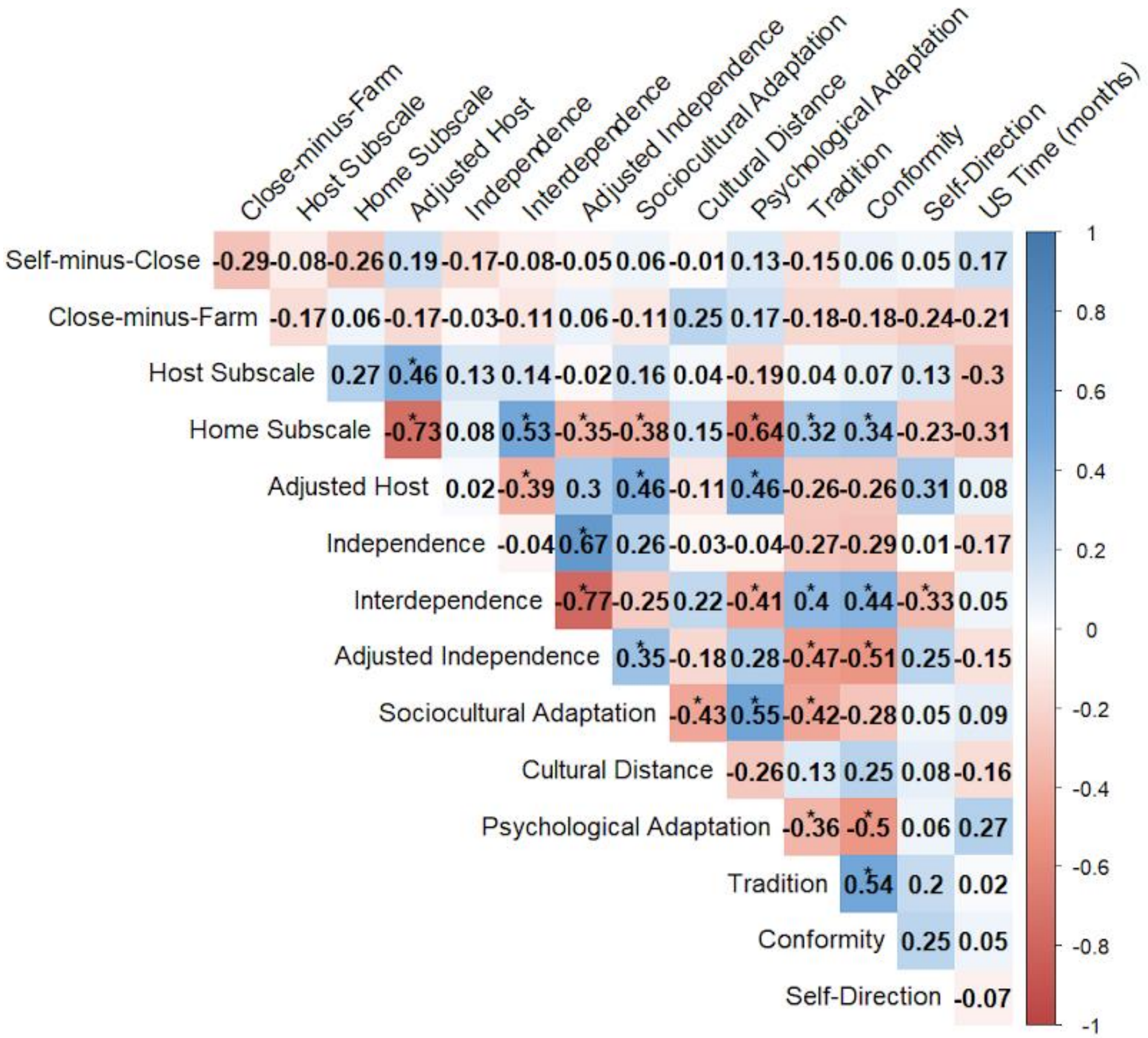

There were no significant correlations between any potential measures of acculturative experience (psychological adaptation, perceived cultural distance, sociocultural adaptation) or relevant cultural values (tradition, conformity, self-direction) with our outcomes of interest (measures of self-referencing in memory: self minus close; close minus farm). Many of the questionnaire measures were highly correlated with each other and with our primary predictor variables of interest (acculturation: AOS host and home; self-construal: independence and interdependence).

For example, the primary predictor of adjusted host score was positively correlated with both sociocultural adaptation ($r = 0.46$) and psychological adaptation ($r = 0.46$), and both of those items were positively correlated with each other ($r = 0.55$). Similarly, the primary predictor of adjusted independence was negatively correlated with both conformity ($r = -0.51$) and tradition ($r = -0.47$), and both items were positively correlated with each other ($r = 0.54$).

Because many of the scales were related to each other, we conducted an exploratory factor analysis (EFA) to assess whether these measures would provide additional information if included in models. The EFA (n = 39) included nine items: adjusted host score, adjusted independence, time in the US, sociocultural adaptation, psychological adaptation, perceived cultural distance, and the cultural values of conformity, tradition, and self-direction. Z-scores were used and scores were reversed as necessary, so all scales were coded in the direction of a higher score indicating more American-like responses or greater ease in adapting to the United States. Eigenvalues, a scree plot, and parallel analysis were used to determine the number of potential factors. Eigenvalues suggested a 3-factor solution whereas the scree plot and parallel analysis suggested a 1-factor solution. 3, 2, and 1-factor solutions were then examined, testing which best fit the data using a Promax rotation. 3 and 2 factor solutions were not sufficient ($p = 0.80$, $p = 0.50$). However, a 1-factor solution was sufficient ($X^2(27, N = 39) = 42.83$, $p = 0.03$. This 1-factor solution explained 29% of the variance. The 1-factor solution results suggested that adjusted host score, adjusted independence, sociocultural

adaptation, psychological adaptation, conformity, tradition, and perceived cultural distance loaded onto a single factor.

Loadings on factor 1:

Adjusted Host	0.54
Adjusted independence	0.57
Sociocultural adaptation	0.69
Psychological adaptation	0.71
Conformity	0.65
Tradition	0.62
Perceived cultural distance	0.38

Items that did not load onto a factor included US time and self-direction. If we examine the scale reliability when including only the seven measures listed above, Cronbach's alpha is 0.78 and mean inter-item correlation is 0.35. Dropping perceived cultural distance, which has the lowest loading on factor 1, increases Cronbach's alpha to 0.80. Dropping any other items only decreases alpha.

These results suggest that the additional questionnaires measure the same overall construct as acculturation, the primary measure that was the focus of the original submission. For this reason, as well as the sample size (i.e., N = 92 for the primary analyses with acculturation vs. 39 for the complete set of measures), we report the results with acculturation in the main text and these additional analyses here. In future research, it may be useful to combine these measures into a single composite measure of acculturation, using confirmatory factor analysis to ensure this is a meaningful and reliable measure.
